# Formyl-Peptide Receptor Agonists and Amorphous SiO_2_-NPs Synergistically and Selectively Increase the Inflammatory Responses of Human Monocytes and PMNs

**DOI:** 10.5772/62251

**Published:** 2016-01-01

**Authors:** Regina Tavano, Daniela Segat, Chiara Fedeli, Giulia Malachin, Elisa Lubian, Fabrizio Mancin, Emanuele Papini

**Affiliations:** 1 Department of Biomedical Science, University of Padua, Padua, Italy; 2 Department of Chemical Science, University of Padua, Padua, Italy

**Keywords:** Silica Nanoparticles, Monocytes, PMNs, Formyl Peptide Receptors, Immunomodulation

## Abstract

We tested whether amorphous SiO_2_-NPs and formylpeptide receptor (FPRs) agonists synergistically activate human monocytes and neutrophil polymorphonuclear granulocytes (PMNs). Peptide ligands specifically binding to FPR1 (f-MLP) and to FPR2 (MMK-1, WKYMVM and WKYMVm) human isoforms did not modify the association of SiO_2_-NPs to both cell types or their cytotoxic effects. Similarly, the extent of CD80, CD86, CD83, ICAM-1 and MHCII expression in monocytes treated with SiO_2_-NPs was not significantly altered by any FPRs agonist. However, FPR1 stimulation with f-MLP strongly increased the secretion of IL-1β, IL-6 and IL-8 by human monocytes, and of IL-8 by PMNs in the presence of SiO_2_-NPs, due to the synergic stimulation of gene transcription. FPR2 agonists also up-modulated the production of IL-1β induced by monocytes treated with SiO_2_-NPs. In turn, SiO_2_-NPs increased the chemotaxis of PMNs toward FPR1-specific ligands, but not toward FPR2-specific ones. Conversely, the chemotaxis of monocytes toward FPR2-specific peptides was inhibited by SiO_2_-NPs. NADPH-oxidase activation triggered by FPR1- and FPR2-specific ligands in both cell types was not altered by SiO_2_-NPs. Microbial and tissue danger signals sensed by FPRs selectively amplified the functional responses of monocytes and PMN_S_ to SiO_2_-NPs, and should be carefully considered in the assessment of the risk associated with nanoparticle exposure.

## 1. Introduction

Cytotoxicity and inflammation are dangerous adverse events that may be associated with nanomedical applications[1]. Indeed, many nanosystems induce cell death and an increased synthesis of cytokines and chemokines, both in vitro and in vivo [2–4]. Amorphous silica is considered more biocompatible than highly toxic crystalline silica and has therefore been proposed as a nanomaterial [5]. However, amorphous nanosilica has cytocidal and pro-inflammatory properties as well, including the ability to induce the secretion of powerful cytokines by myeloid cells such as IL-1β and TNFα. In vitro studies indicate that toxicity and cytokines synthesis are triggered by SiO_2_-NPs above a threshold concentration. In addition, inflammatory immune cells like monocytes and macrophages are about 10 times more sensitive to the toxic action of SiO_2_-NPs than non-phagocytic leukocytes and epithelial cell lines [6]. This higher sensitivity of monocytes/macrophages to amorphous SiO_2_-NPs is likely due to their higher capturing efficacy, leading to NPs' intracellular accumulation up to a critical level and determining cell death [6]. Available evidence indicates amorphous silica particles induce maturation and secretion of IL1β in monocytes and macrophages by activating caspase-1 [6–8]. Recent data suggest that the activation of caspase-1 by SiO_2_-NPs also mediates pyroptotic, pro-inflammatory cell death in monocytes, a phenomenon that may further exacerbate tissue inflammation [6].

However, it is possible that the adverse reactions to SiO_2_-NPs and their intensities are only partially estimated, based on these in vitro evidence. Other factors generated in vivo may synergize with the action of NPs. In particular, costimulation with microbial agonists (PAMPs) may contribute to the activity of nanomaterials. For example, LPS, the major outer membrane component of Gram- bacteria, has been long known to upregulate the ability of nano-micro particulates to induce IL1-β secretion in macrophages and monocytes [6–9]. While macrophage endocytosed nano-micro particulates eventually activate caspase-1, LPS stimulates the NFK-b dependent transcription of pro-IL1β. Some evidence also suggests that LPS may improve the cytotoxic effect of nanoparticles [9]. In addition, f-MLP, a prototype of prokaryotic-derived formylated leader sequences, synergizes the induction of various cytokines by both naked and PEGylated ORMOSIL-NPs, liposomes and PLGA-NPs [10]. N-formylated peptides bind to a group of seven-transmembrane-domain Gi protein-coupled receptors (GPCR), called FPRs (formyl peptide receptors), which are expressed by myeloid cells. Nanomolar or sub-nanomolar concentrations of FPRs-specific ligands are a potent alarm signal for inflammatory and immune cells, indicating the presence of an infection. The activation of FPRs determine a multiple response, aimed at recruiting more defensive inflammatory cells in the infected tissue and the elimination of invading microbes. These FPR-mediated effects include intracellular calcium mobilization, the activation of antimicrobial mechanisms like NADPH oxidase and the degranulation and stimulation of chemotaxis. In humans, three FPR homologues are known: FPR1, FPR2 and FPR3. FPR1 binds with high affinity (Kd in the nM range) to N-fMLP and other N-formylated peptides from *L. monocytogenes* [11–13]. FPR2 binds with low affinity to f-MLP (Kd in the μM range), but with nanomolar affinities to several microbial derived molecules (the peptide Hp(2–20) from *H. Pylori* [14], formylated peptides from *L. monocytogenes* and staphylococcal phenol-soluble modulins [15]). Interestingly, a set of various host-derived peptides and molecules also bind to FPR2: annexin-derived Ac1–25, the antimicrobial peptide cathelicidin LL37, a fragment of the urokinase receptor, serum amyloid A, amyloid b peptide (Aβ42), the prion protein fragment PrP(106–126) [14] and the anti-inflammatory lipid mediator lipoxin A4 [16]. The binding specificity of FPR3 is the least characterized of the three human isoforms. It does not bind to N-fMLP, but specifically associates with the acetylated N-terminal peptide in human haem-binding protein [17]. FPR1 and FPR2 are both expressed in PMNs and monocytes, while FPR3 is only present in monocytes and DCs [14].

The above summarized FPRs specificities suggest that while FPR1 monitors bacterial infections, FPR2, due to its promiscuous binding capability, is sensitive to both bacterial presence and tissue alterations. However, such a functional distinction between FPR1 and FPR2 is not clearcut, because, for example, formylated peptides from mitochondrial proteins (like NADH dehydrogenase and Cox subunits) bind to both FPR1 and FPR2 with similar high affinities (Kd range 12–210 nM) [13]. Since mitochondria-derived formyl-peptides are released into the extracellular space during necrosis, FPR1 likely also plays a crucial role in the recruitment and activation of leukocytes in pathologies characterized by sterile inflammation and tissue damage [18]. A recent analysis showing that FPRs can recognize common structural motives present in hundreds of microbial and tissue peptides supports the hypothesis that this versatile receptor family has a central homeostatic role in mammals by monitoring their microbioma [19].

Some synthetic peptides identified in random peptide libraries are valuable tools for studying the function of different FPRs isoforms. The MMK-1 peptide, which has a sequence of LESIFRSLLFRVM [20], is a FPR2 specific agonist able to induce Ca_2_^+^ mobilization in FPR2-transfected cells with an EC50 of around 2 nM [21]. Similar effects on cytosolic calcium were observed in monocytes and PMNs in the same nmolar range. MMK-1 also induces chemotaxis with a maximum at about 1 μM in both monocytes and PMNs and NADPH-oxidase dependent ROS [22]. WKYMVM activates FPR2-transfected HL60 cells with an EC50 of about 2 nM and FPR3-transfected ones with an EC50 of around 80 nM, but does not stimulate FPR1-expressing cells [23]. WKYVMVm binds to all FPR isoforms with different affinities: at picomolar concentrations to FPR2 (Kd 75 pM) and in the nanomolar range to FPR3 (Kd 3 nM) and to FPR1 (Kd 25 nM). Consequently, WKYMVm is the most effective FPRs agonist able to induce chemotaxis and NADPH-oxidase activation in PMNS and monocytes at very small doses [24].

In synthesis, FPRs may be considered unique PRR receptors, able to sense both PAMPs and DAMPs. For this reason, in this study, we decided to examine the possibility that nanoparticles act synergistically with FPRs' activation. Human PMNs and monocytes were incubated using the nanosystem amorphous silica model, with NPs in the absence or in the presence of the FPR specific peptides, as summarized in Table 1.

**Table 1.** Expression of formyl peptide receptors on human PMNs and monocytes and the affinity (K_d_) of different FPR agonists for different FPR isoforms [14]

	FPR1	FPR2 (FPR1-L)	FPR3 (FPR2-L)
PMNs	+	+	−
Monocytes	+	+	+
f-MLP	1–3 nM	1 μM	No binding
MMK-1	No binding	2 nM	10 μM
WKYMVM	No binding	2nM	80 nM
WKYMVm	25 nM	0.075 nM	3nM

SiO_2_-NPs doses and incubation times (generally 50 and 100 μg/ml for 24 hours) were calibrated based on our previous study [6] and showed that both Ludox and Stöber silica NPs (diameter 24 and 27nm, respectively) had intrinsic pro-inflammatory and cytotoxic effects on monocytes in these conditions.

Cytotoxicity, superoxide anion induction, overexpression of CD markers involved in antigen presentation, cytokine production and chemotaxis were analysed. Results indicate a reciprocal interaction between FPR activation and SiO_2_-NPs action on both PMNs and monocytes, eventually leading to a stronger inflammogenic response from these cells.

## 2. Material and Methods

### 2.1 Nanoparticles

LUDOX® TM40 colloidal silica nanoparticles (NPs) (40% wt. suspension in H_2_O) were purchased from Sigma Aldrich. Nanoparticles' DLS measurements provided an average diameter of 28 nm with a 0.205 PDI (in phosphate-buffered saline [PBS], pH 7.4). The zeta-potential value (same conditions as DLS analysis) was −13.7 mV and TEM analysis yielded a 27 nm diameter in good agreement with the DLS size. All NPs suspensions were endotoxin free (<0.05 Endotoxin units/ml, as measured by a Limulus test). Stöber nanoparticles were prepared as previously described [6]. DLS measurements provided an average diameter of 30 nm with a 0.198 PDI (in phosphate-buffered saline [PBS], pH 7.4); zeta-potential value (same conditions as DLS analysis) was −18.8 mV and TEM analysis yielded a 24 nm diameter.

### 2.2 Purification of monocytes and PMN

Peripheral blood mononuclear cells (PBMC) were isolated from the buffy coats of healthy donors by centrifugation over a Ficoll-hypaque (Amersham Biosciences) step gradient and a subsequent Percoll (Amersham Biosciences) gradient, and suspended in RPMI-1640 (GIBCO BRL) supplemented with antibiotic. Residual T and B cells were removed from the monocyte fraction by plastic adherence for 1 hour at 37°C. The purity of preparations (percentage of CD14-positive cells) and cell viability (using the trypan blue exclusion test) were both higher than 98%. For human PMNs purification, after centrifugation of buffy coats through a Ficoll-hypaque gradient, red blood cells contained in the bottom fraction were eliminated by dextran sedimentation, followed by hypotonic lysis and washing with PBS. The percentage of contaminating cells was <5%. Unless otherwise specified, monocytes and PMNs were kept at 37°C in a humidified atmosphere containing 5% (v/v) CO_2_ in RPMI-1640 and supplemented with antibiotic and 10% FCS (Euroclone).

### 2.3 Measurement of IL-1β, IL-6 and IL-8 production by monocytes and PMNs

2×10^6^ Monocytes or PMNs were incubated with different concentrations (50-100-200 μg/ml) of NPs and in presence or absence of different bacterial stimuli (fMLP from Sigma Aldrich, MMK1, WKYMVM and WKYMVm from Tocris Bioscience) for 20h at 37°C. Culture supernatants were collected and the amount of IL-1β, IL-6 and IL-8 protein was quantified by ELISA assay, following the manufacturer's instructions.

### 2.4 Real-time PCR analysis

2×10^6^ Monocytes were incubated for five hours at 37°C, as previously described. Treated and untreated cells were scraped and total RNAs were isolated using a TRIzol solution (SIGMA), according to the manufacturer's instructions, and suspended in 12 μl of RNase-free water (Gibco). RNA was quantified by spectrophotometric analysis (NanoDrop® ND-1000 Spectrophotometer, CELBIO). Equal amounts of RNA (300 ng) was retrotranscripted and the concentration of cDNA for IL-1β, IL-6, IL-8 and TNF-α were quantified by real time quantitative PCR using an iQTM SYBR Green Supermix (Biorad) and an iQ5 2.0 Biorad System, according to the manufacturer's instructions (Biorad). The following primers were used: for GAPDH, 5′-AGCAACAGGGTGGTGGAC-3′ and 5′-GTGTGGTGGGGGACTGAG-3′; for IL-1β, 5′-CTGTCCTGCGTGTTGAAAGA-3′ and 5′-TTGGGTAATTTTTGGGATCTACA-3′; for IL-6, 5′-AACCTGAACCTTCCAAAGATGG-3′ and 5′-TCTGGCTTGTTCCTCACTACT-3′; for IL-8, 5′-TTGGCAGCCTTCCTGATT-3′ and 5′-AACTTCTCCACAACCCTCTG-3′; for TNF-α, 5′-ATGAGCACTGAAAGCATGATC-3′ and 5′-GAGGGGCTGATTAGAGAGAGGT-3′.

Each run was completed with a melting curve analysis to confirm the specificity of amplification and lack of primers' dimers. CT (cycle threshold) values were determined by the GeneAmp 5700 SDS software using fluorescence threshold, manually set and exported into Excel for analysis. Following the amplification, data analysis was performed using the second derivative method algorithm. For each sample, the amount of messenger RNA (mRNA) of the cytokines was expressed as the n-fold of the normalized amount of mRNA in untreated cells (1 arbitrary unit = cytokine mRNA concentration/GAPDH mRNA concentration [both in fmoles/μl]).

### 2.5 Measurement of marker expression by flow cytometry analysis

Monocytes (2×10^6^) were incubated in the presence of different bacterial stimuli for 20 hours with 50 μg/ml of NPs in a RPMI-1640 medium, supplemented with 10% FCS at 37°C. Cells incubated with no nanoparticles were used as a negative control. Cells were then washed with phosphate-buffered saline pH 7.2, scraped, centrifuged, resuspended in a FACS buffer (PBS containing 1% FCS and 0.1% NaN_3_) and then further incubated with the proper dilution of different anti-CD monoclonal antibodies (CD80, CD83, CD86, ICAM-1 and MHC-II, Biolegend), and conjugated to phycoerythrin (PE) for 30 minutes on ice. After a wash with the FACS buffer, propidium iodide was added to exclude dead cells and the cell fluorescence intensities of the gated populations were measured with a FACS Canto flow cytometer, and analysed using FACSDiva software (Becton Dickinson).

### 2.6 NPs association to monocytes and PMNs

Monocytes and PMNs (2×10^6^), seeded onto 24-well plates, were incubated with different concentrations (up to 200 μg/ml) of Stöber NPs for 24 hours in a RPMI-1640 medium, supplemented with 10% FCS, at 37°C, in the presence or absence of bacterial stimuli. Then cells were collected, washed with PBS and suspended in a cold FACS buffer (PBS containing 1% FCS). Propidium iodide was added to exclude dead cells. Nanoparticles' capture was evaluated as mean florescence intensity (MFI) intensities of the gated populations, using a BD FACSCanto II flow cytometer and FACSDiva software (Becton Dickinson).

### 2.7 MTS cytotoxicity assay

Monocytes (1 × 10^6^ cells/ml) were plated into a 96-well culture plate the day before the experiment. Cells were then incubated for 18h with Ludox TM40 NPs at different concentrations (up to 200 μg/ml) in a complete medium and with bacterial stimuli. Cellular mitochondrial activity (an indicator of cellular viability) was evaluated by MTS assay (Promega), according to the instruction manual.

### 2.8 Chemotaxis assay

Monocytes or PMNs were purified and immediately seeded in the upper chamber of a Transwell plate (Corning) in a complete medium (0.1×10^6^ per condition). The lower chambers were filled with complete medium only, or with complete medium containing the different bacterial stimuli at two concentrations (1 nM and 1 μM), either in presence or absence of 100 μg/ml of SiO_2_-NPs. After 2h at 37 °C, the number of monocytes or PMNs that had migrated into the lower chamber was estimated by BD FACSCanto II flow cytometer.

### 2.9 Superoxide anion production

The cell release of O_2_^.-^ was estimated by cytochrome c reduction assay. Briefly, PMNs were incubated at 37°C in the presence or absence of 100 μg/ml NPs and different concentrations of bacterial stimuli in HBSS pH 7.4 containing 80 μM ferricytochrome c type III (Sigma). Cytochrome C reduction was evaluated at 550 nm at different time points using an automated microplate reader (Multiscan Go, Thermo Scientific).

## 3. Results

**Figure 1.**
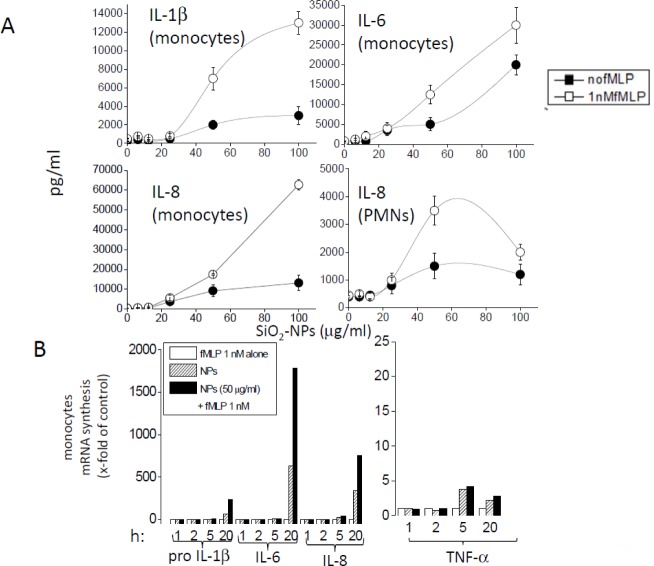
**The effect of f-MLP on the production of proinflammatory cytokines and chemokines induced by SiO_2_-NPs in monocytes and PMNs**. A) Dose response of IL-1β, IL-6 (monocytes) and IL-8 (monocytes and PMNs) induction by SiO_2_-NPs in the absence and in the presence of 1 nM f-MLP. Data are the mean ± SE (N=3); B) Representative RT-PCR analysis, out of three, of the kinetics of the transcription of *preIL-1β*, *IL-6*, *IL-8* and *TNFα* genes in monocytes treated with f-MLP or NPs only, or in combination, as indicated; *h* indicates the incubation time expressed in hours. mRNA production is expressed as multiples of the value measured in cells treated with f-MLP only after 1 hour.

**FPR1 and FPR2 activation upregulates the efficacy of SiO_2_-NPs to induce cytokines/chemokines release by monocytes and PMNs**. We first characterized the effect of f-MLP on the intensity of cytokine and chemokine production by monocytes at a concentration (1 nM) only occupying FPR1, and by PMNs treated with SiO_2_-NPs. Although f-MLP was inactive per se, it further increased the intrinsic efficacy of SiO_2_-NPs to induce cytokines and chemokines in both monocytes and PMNs, above the NPs' dose threshold of 25 μg/ml [6] (Fig. 1A). RT-PCR analysis (Fig. 1B) indicated that f-MLP increased the synthesis of IL1β, IL-6 and IL-8 induced by SiO_2_-NPs (50 μg/ml) in monocytes, but not that of TNFα, by synergizing the transcription of the mRNA encoded by *preIL-1β*, *IL-6* and *IL-8* genes. Since the transcription rate of these genes are under the control of NF-kB, the data strongly suggest that f-MLP and SiO_2_-NPs synergic action is mediated by this transcription factor. Consistently, western blot analysis with specific antibodies indicated an increased expression of NF-kB in monocytes after 3.5h co-stimulation with both FPR1- and FPR2- specific agonists and SiO_2_-NPs, compared to the expression observed in control cells or in cells treated with the peptide or nanoparticles only (data not shown).

**Figure 2.**
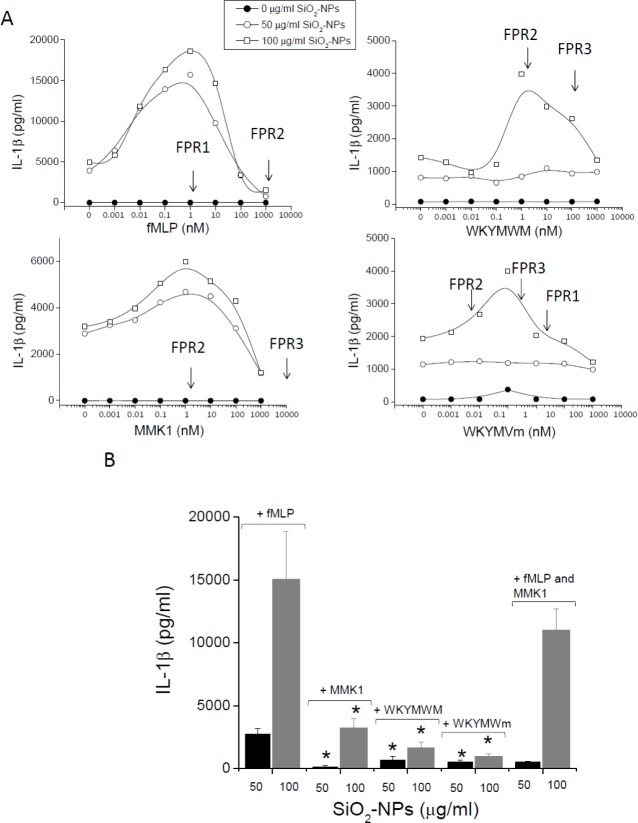
**The effect of f-MLP, MMK-1, WKYMVM and WKYMYm on the production of IL1β by monocytes in the presence of SiO_2_-NPs**. A) Representative dose-response curves (out of 10) of the effect of the indicated peptides on the cytokine extracellular release after 24 h, measured by ELISA assay, in the presence of the indicated NPs concentrations. Within each panel, arrows point to the peptide concentrations approximately corresponding to the Kd values characterizing interaction with the indicated FPR isoforms (see also Tab. 1). B) IL1β secretion by monocytes incubated with SiO_2_-NPs at the indicated doses for 24h in the presence of the indicated peptides (1 nM). Data are the mean ± SE (N=10). * p<0.05 with respect to f-MLP treated cells.

Having observed the ability of the FPR1 agonist f-MLP to modulate SiO_2_-NPs activity, we tested and compared the action of other peptides binding to the different FPR isoforms. Dose-response analysis (Fig. 2A) of the effect of f-MLP on IL-1β released by monocytes stimulated with SiO_2_-NPs also confirmed the binding to FPR1 receptors on these cells to be responsible for the observed synergy. In fact, cytokine production reached a maximum at peptide doses (1–3 nM) corresponding to the binding affinity (Kd) of f-MLP to this receptor. Contrarily, similar to what was observed in the case of ORMOSIL-NPs [10], this synergy almost dropped to zero at f-MLP concentrations (∼ 1 μM), saturating FPR1 and partially titrating FPR2. This biphasic curve may suggest that the occupation of FPR2 receptors by f-MLP determines an inhibitory signal, contrasting that generated by FPR1. Alternatively, high doses of f-MLP may rapidly induce the downregulation of FPR1. Experiments performed with the peptides MMK1 and WKYMVM unable to bind to FPR1, but associating with high affinities to FPR2 showed that activation of this receptor isoform also synergized the production of IL-1β by monocytes treated with SiO_2_-NPs. Moreover, also in this case, the peptide effect reached a maximum in correspondence with the FPR2 Kd (2 nM) and decreased at higher concentrations (>10–100 nM). In the case of MMK-1 co-stimulation, this decrease of cytokine synthesis could not be ascribed to the binding to other low affinity receptors. In fact, the affinities of MMK-1 for FPR3 and FPR1 was extremely low (Kd=10 mM) or undetectable, respectively. The ability of activated FPR2 to mediate an increased production of IL-1β was confirmed by the agonist WKYMVm. This peptide, characterized by the presence of a D methionine at the C terminal, is known to bind with a very high affinity to the FPR2 (Kd=75 pM) and with relatively minor affinities to FPR3 (Kd=3 nM) and FPR1 (Kd=25 nM). Consistently, the WKYMVm effect was detected at concentrations ensuring significant and specific occupation of FPR2 (10–100 pM). Hence, data collectively indicate that both FPR1 and FPR2 isoforms synergize with SiO_2_-NPs to induce a strong secretion of IL-1β by monocytes. However, quantitative comparisons of data obtained using cells from several independent donors (N=10) indicate that the FPR1-mediated effect is three times stronger than the FPR2-mediated effect (Fig. 2B). Eventually, the co-incubation of cells with both fMLP and MMK1 determined a cytokine secretion not dissimilar to those induced by single agonists in the presence of SiO_2_-NPs, suggesting no reciprocal interference between FPR1- and FPR2-mediated signals. The ability of FPR3 to mediate similar synergic phenomena in the presence of SiO_2_-NPs could not be established due to a lack of specific ligands.

**FPRs activations did not increase SiO_2_-NPs cellular capture and cytocidal effects**. A possible mechanism that can simply explain the synergy of the FPR agonist to boost SiO_2_-NPs' ability to induce IL-1β may be the induction of a higher ability to capture nanoparticles and/or to a stronger sensitivity of cells to the cytotoxic action of SiO_2_-NPs. Indeed, f-MLP may induce the overexpression of scavenger receptors mediating silica NPs' binding, thereby improving the nanoparticle load and their cytotoxic effect. Moreover, cytotoxicity is well-known to be strictly linked to the secretion of cytokines [6].

**Figure 3.**
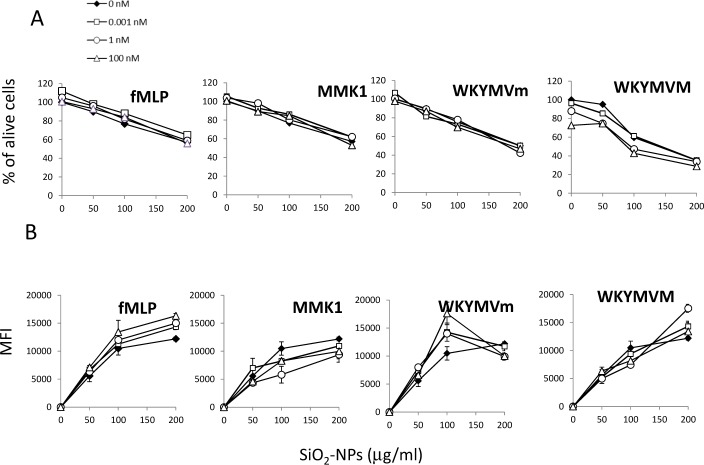
**Effect of FPR agonists on SiO_2_-NPs monocytes capture and on SiO_2_-NPs induced cytotoxicity**. A) Cells were incubated for 24h with SiO_2_-NPs alone or in the presence of peptides, as indicated and washed and subjected to MTS assay. Values are represented as percentages of alive cells in control cells (no NPs and no peptide). Data are the mean from three independent experiments run in triplicate. Error bars (± SE), omitted for representation clarity, never exceeded ± 15%. Differences of values in the presence of peptides with respect to values in the absence of peptides were not statistically significant. B) Cells, incubated for 24h with fluorescein-labelled SiO_2_ Stöber-NPs and the indicated peptide doses were washed and the fluorescence associated with cells was measured by FACS and expressed as mean fluorescence intensity (MFI). Values are means ± SE (N=3).

However, the association of FITC labelled SiO_2_-NPs to monocytes and to PMNS (not shown) was not significantly modulated by any of the used peptides, and, consistently, cell death extent induced by SiO_2_-NPs was not improved by FPR1/FPR2 agonists in the full dose range used (0.01–1000 nM) (Fig. 3).

**Figure 4.**
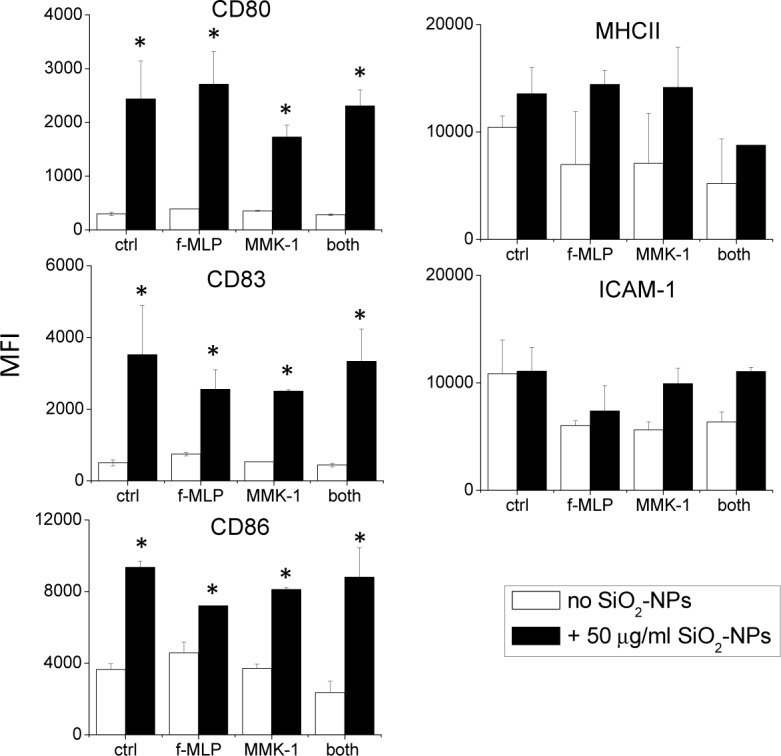
**The effect of FPR agonists on the expression of immunological activation markers in monocytes treated with SiO_2_-NPs**. Monocytes were incubated for 24h in the presence of NPs and peptides, as indicated. Cells were then scraped, incubated with different antibodies to the medicated CD markers and the fluorescence associated with cells was measured by FACS. Data are means ± SE (N=4), * p<0.05 with respect to untreated cells.

**FPRs activations did not synergize with SiO_2_-NPs in the upregulation of antigen-presentation-related proteins in monocytes**. The selectivity of FPR1 and FPR2 agonists in increasing the production of proinflammatory cytokines/chemokines was confirmed in other experiments where we analysed the expression of CD80, CD83, CD86, MHCII and ICAM-1 (Fig. 4). These membrane proteins are involved in antigen presentation and T-lymphocytes' co-stimulation and their overexpression indicated the induction of the immunological competence of these cells. Interestingly, SiO_2_-NPs determined the over expression of some of these markers (CD80, CD83, CD 86) but not that of others (MHC-II and ICAM-1). However, in no cases did f-MLP and MMK-1, alone or in combination, synergize a statistically significant over-expression of the level of CD80, CD83, CD86 and ICAM-1 induced by SiO_2_-NPs. MHC II expression appeared to be increased by SiO_2_-NPs co-incubated with peptides, but this trend was not statistically significant. Overall, data indicated that FPR1 and FPR2 activation, alongside an increase in the production of inflammatory cytokines, do not stimulate the adaptive immunological functions of monocytes.

**Figure 5.**
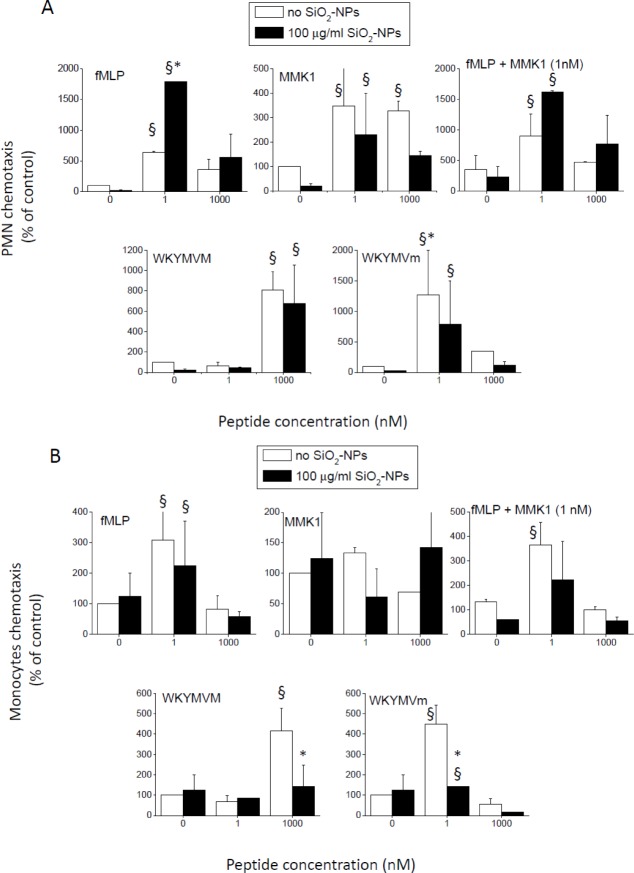
**The effect of SiO_2_-NPs on the chemotactic migration induced by FPRs' ligands in PMNs and monocytes**. PMNs (A) and monocytes (B) were seeded in the upper chamber of Transwell plates, in RPMI plus 10% FCS at 37°C, while peptides at the indicated doses and NPs (100 μg/ml) were present in the lower chambers in the same medium. After two hours, the cells that had migrated into the lower chambers were harvested and counted by FACS. (Data are means ± SE (N=4); § p<0.05 with respect to control cells; * p<0.05 with respect to cells treated with the same concentration of FPR agonist, but in the absence of silica nanoparticles.

**SiO_2_-NPs differentially modulated the chemotactic migration induced by FPR1- and FPR2-specific peptides in PMNs and monocytes, but had no effect on the activation of NADPH oxidase triggered by the same agonists**. In the previous paragraphs, we have shown how FPR1- and FPR2-selective peptides further increased the synthesis of cytokines and chemokines induced by SiO_2_-NPs in monocytes and PMNs. We then decided to verify whether, in turn, SiO_2_-NPs modulated the major pro-inflammatory effects mediated by FPRs agonists, that is, chemotaxis and the activation of the NADPH-oxidase responsible for the production of superoxide anion. In agreement with previous studies [14,25–30], all used peptides stimulated the chemotaxis of both PMNs and monocytes. Interestingly, SiO_2_-NPs, although inactive when tested as single agonists, significantly increased the chemotactic migration of PMNs induced by f-MLP (FPR1) but did not modify the one triggered by FPR2-specific agonists (Fig. 5A). Even in this assay, as in the case of IL1β production, high doses of f-MLP failed to synergize with nanoparticles. Once again, this was due not to the activation of FPR2 by f-MLP occurring at micromolar doses, but was instead likely the consequence of rapid receptor downregulation. In fact, cells' co-stimulation with SiO_2_-NPs and a mixture of f-MLP (1 nM) and FPR 2-specific MMK 1 (1 nM) did not affect the chemotaxis synergistic effect. Monocytes' chemotaxis toward FPR1 and FPR 2 agonists was, on the contrary, not affected or even inhibited by SiO_2_-NPs (Fig. 5B).

**Figure 6.**
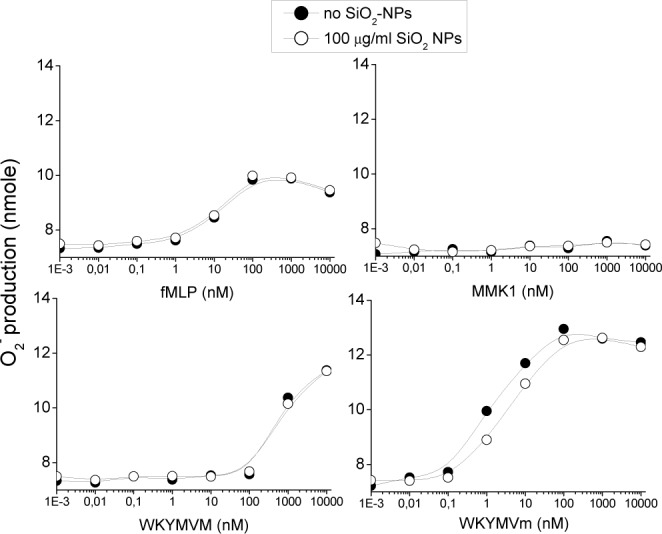
**The effect of SiO_2_-NPs on the respiratory burst induced by FPRs agonists in PMNs**. PMNs were seeded onto 96-well plates and stimulated with SiO_2_-NPs (100 μg/ml) alone or together with the indicated concentrations of f-MLP, MMK1, WKYMVM and WKYMVm in RPMI, plus 10% FCS at 37°C. After 10 minutes, O_2_^.-^ release was estimated spectrophotometrically by cytochrome c reduction assay at 550 nm at different time points. The data given shows a representative graph out of five for each peptide.

Eventually, we tested the possible effect of SiO_2_-NPs on the production of O_2_^.-^ by the NADPH-oxidase induced by FPR agonists. Once again, in agreement with reported evidence [25, 27–30], both FPR1 and FPR2 agonists stimulated rapid activation of the respiratory burst in PMNs. The FPR2 agonist WKYMVm was the most efficient, having already been active at the maximal level at subnanomolar dose, compatible with its high affinity for the FPR2. F-MLP and WKYMVM followed and MMK-1 was the least effective. These data confirmed that both FPR 1 and FPR2 were able to trigger the typical oxygen dependent antimicrobial repose of PMNs. In monocytes, such activation is significantly reduced (not shown). The co-incubation with SiO_2_-NPs, unable to trigger the activation of the NADPH oxidase per se, does not affect or improve the kinetics and dose response of the peptide-induced superoxide production in PMNs (Fig. 6).

## 4. Discussion

Understanding and avoiding adverse reactions induced by nanoparticles is a central aspect of nanomedicine and nanotoxicology. The standard in vitro toxicological approach consists of the exposure of appropriate model cells to purified nanoparticles in a characterized cell culture medium. However, the biological effects of nanosystems are possibly modulated by the presence of agonists generated only in the host tissues and organs. This may be especially critical in the presence of microbial infections or in inflammatory conditions characterized by the presence of factors from both infectious agents and damaged cells. These molecules can potentially tilt the balance of nanoparticles' action toward toxic-pathogenic effects. Indeed, several microbial derivatives are known to mediate or synergize the typical signs of cytotoxic effects, such as ROS overproduction, or may increase the efficacy of other proinflammatory stimuli. On the other hand, tissue-derived molecules, released as a result of cell damage, (e.g., ATP or mitochondrial peptides) are also considered major regulators of inflammatory and immune responses, both in physiological and pathological processes. In this study, we found manifold evidence that microbial (PAMPs) and damaged tissue molecular patterns (DAMPs) could, in principle, critically increase the bio-hazardous nature of nanosystems. In fact, we documented a synergistic effect between amorphous SiO_2_-NPs, known to determine an inflammatory and cytotoxic effect both in vitro and in vivo [6,7,9], and specific agonists for FPRs, a receptor family specialized in monitoring both bacterial-derived and cytosolic-derived signals. To do this, we exploited the well characterized peptides f-MLP (specific to the FPR1 human isoform in the namolar concentration range), MMK-1 (FPR2-specific in the nanomolar range), WKYMVM (FPR2-and FPR3-selective) and WKYMVm (binding with very high affinity to FPR2). We analysed the ability of these FPR agonists to modulate the intrinsic ability of SiO_2_-NPs to trigger cell death and cytokine production in immune cells. In addition, we examined whether chemotaxis and NADPH-oxidase activation triggered by FPR agonists in monocytes and PMNs were modulated by SiO_2_-NPs.

**Figure 7.**
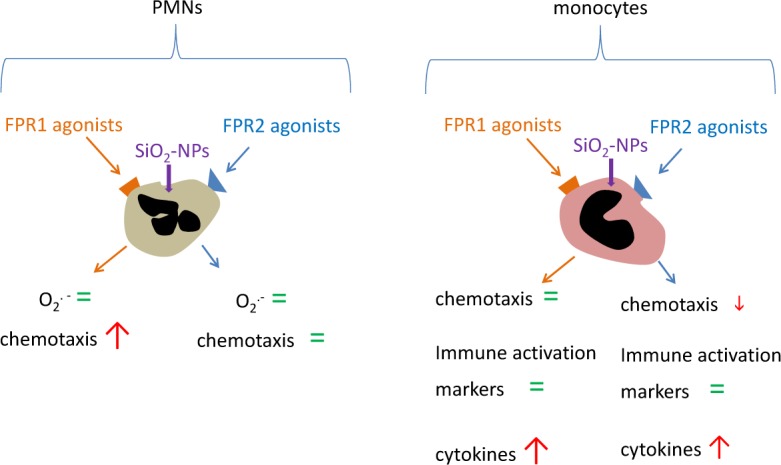
Model of the interaction between nanoparticles and monocytes/polymorphonuclear leukocytes in activating defensive and immune response. In PMNs, silica nanoparticles do not induce modulation of the oxidative burst induced by FPR agonists. Silica nanoparticles induce a stronger chemotaxis when co-incubated with FPR1 agonists, but not with FPR2 agonists. On the other hand, in monocytes, the co-incubation of both FPR agonists with silica nanoparticles induce the strong modulation of cytokine release, leaving the expression of different activation markers unaffected. Furthermore, FPR2-dependent chemotaxis, but not FPR1-dependent chemotaxis, decreased in the presence of silica nanoparticles.

FPR agonists did not increase the cell sensitivity to NPs-induced acute toxic effects. However, FPR1 and FPR2 activation increased the production of cytokines, and especially of the master inflammatory signal IL-1β in the presence of amorphous silica NPs, with significant effects detectable at peptide concentrations as low as ∼ 10 pM. The chemotactic migration of PMNs toward nanomolar concentrations of the FPR1 agonist f-MLP, but not toward FPR2 specific peptides, was synergistically increased by the presence of NPs. On the contrary, the chemotaxis of monocytes toward all tested peptides was not increased by NPs' co-treatment, but instead inhibited (Fig. 7). This inhibitory effect of NPs on the chemotaxis of monocytes induced by peptides may have been due to sequestration of the peptide by NPs. However, since in PMNs, at least in the case of f-MLP, NPs increase chemotaxis, this eventuality will not compromise the final functional stimulation of cells.

Collectively, our data suggest that the proinflammatory effects induced by amorphous silica in vivo may become, in defined circumstances, much stronger than has thus far been estimated in vitro. In fact, the presence of even small concentrations of FPRs-specific molecules released by either pathogenic or commensal bacteria could exacerbate the proinflammatory effects of amorphous SiO_2_-NPs and possibly of other nanoparticles mediated by myeloid cells. A similar synergistic effect could also be mediated by necrotic cells following either infective or sterile tissue damage. In fact, mitochondria-derived formyl peptides, normally retained in live cells but released by necrotic or apoptotic dying cells, bind to both FPR1 and FPR2 with comparable affinities. We can speculate that an initial condition of tissue damage typical of many pathologies, microbial infections included, may exacerbate the intrinsic tendency of amorphous silica nanoparticles to trigger inflammatory responses of blood-circulating immune cells, e.g., the PMNs and monocytes tested in this paper. This event will in turn further amplify tissue and organ damage.

The likely human exposure to nanostructured silica nanomaterials, due to its broad use as a food and drug additive, renders the scenario discussed here plausible. This demands the accurate evaluation of amorphous nanosilica proinflammatory/cytotoxic potential, not only in the presence of FPR agonists, but also in the presence of other signals generated by infectious agents, damaged host cells and possibly by commensal microbial flora.

## 5. Conflict of interest

The authors declare no conflict of interest.
